# Optimization Model of Financial Market Portfolio Using Artificial Fish Swarm Model and Uniform Distribution

**DOI:** 10.1155/2022/7483454

**Published:** 2022-06-15

**Authors:** Yao Xiao

**Affiliations:** Chongqing Industry Polytechnic College, Chongqing 400000, China

## Abstract

The central issue in finance is how to select a portfolio in the financial market. The traditional artificial fish swarm algorithm (AFSA) is optimized in this paper, and the improved AFSA is used to solve the portfolio model. This model generates a uniform distribution operator using uniform distribution and combines it with the basic fish swarm algorithm. Uniform variation occurs when the variance of the optimal value of continuous convergence is within the allowable error. In this manner, the fish can escape the trap of the local extremum, obtaining the global optimal state. To validate the feasibility of improving AFSA, this paper conducts simulation experiments on portfolio problems using MATLAB tools. Experiments show that this model has an accuracy of 93.56 percent, which is 8.43 percent higher than that of the NSGA-II model and 3.76 percent higher than that of the multiobjective optimization model. The experiment shows that the algorithm in this paper can solve these types of problems well and that, using this model, the optimal portfolio investment decision scheme satisfying investors can be obtained. The optimized AFSA presented in this paper can serve as an important reference for investment portfolios and has a wide range of application possibilities in the investment market.

## 1. Introduction

With the increasing trend of economic globalization and financial integration, the regional economy has become more market-oriented and transparent. Market competition is all around us, and it is becoming more fierce and diverse [1]. While considering the enormous benefits that investment can bring, business should be prepared for the potential risks that come with it. During the capital investment process, rational investors will consider how to allocate a certain amount of capital to thousands of risky securities in order to spread the risk as widely as possible and obtain the greatest benefit [2]. The securities market, on the other hand, is a financial trading market where risks and interests coexist. Its risk changes are influenced by a variety of factors, including politics, economic fluctuations, policy regulation, and company development, making it difficult to grasp its essential characteristics [3]. Because of the volatile economic environment, the types of risks faced by investors in investment also exhibit diversification and variability, and the investment risk is increasing day by day. Individual investors and financial institutions face a central problem in determining how to allocate resources optimally in an uncertain environment in order to maximize income or minimize risk, that is, how to make portfolio selection in the financial market [4]. With the ongoing development of the market economy, investors from all walks of life are frequently confronted with the portfolio problem when engaging in asset investment activities. Portfolio optimization is extremely important in the financial field and has an incalculable impact on the growth of the market economy. Many scholars are currently concerned about it [5]. In today's volatile market, how to assist investors in effectively avoiding risks has gradually become the focus of portfolio research.

The central issue in finance is how to select an investment portfolio in the financial market. The goal of investors is to maximize benefits in a low-risk environment or to bear the least amount of risk while maximizing returns as much as possible. This establishes the need for investors to evaluate the benefits of securities investment and diversify asset risk as much as possible through portfolio model optimization [6]. Because of their high inspiration, effectiveness, and high matching with computer language, artificial intelligence algorithms have piqued the interest and application of researchers in recent years. AFSA (artificial fish swarm algorithm) is a swarm intelligence algorithm made up of many simple autonomous bodies. Each autonomous body is self-contained and can interact with information in its environment, utilizing a novel bottom-up search concept [7]. AFSA employs a novel concept that distinguishes it from other optimization algorithms. AFSA differs from traditional design and solution methods from the specific implementation of the algorithm to the overall design concept, but it can be well integrated with other traditional algorithms at the same time. AFSA's search process is divided into four stages: foraging behavior, tail chasing behavior, clustering behavior [8], and default behavior. The characteristics of AFSA, such as low initial requirements, strong robustness, strong ability to get rid of local extremum, and global optimization, can bring us an opportunity to solve the portfolio optimization problem. It is precisely because of the superior characteristics of AFSA that AFSA has attracted great interest and extensive attention of scholars from all walks of life at home and abroad since it was proposed. With the deepening of the research on this algorithm, many improved algorithms are applied, and the application field is gradually expanded. Therefore, this paper applies AFSA to the optimization of portfolio model. The innovations of this paper are as follows:

1. The AFSA algorithm is applied to financial market portfolio optimization in this paper, and the original AFSA is improved. The adaptive improvement concept is used in this paper to improve the field of view and step size in the traditional AFSA. The field of view and step size gradually change from large to small to meet the needs of various states of algorithm optimization. Simultaneously, AFSA is integrated with a free search algorithm, allowing it to be applied to data in a portfolio problem to produce a better portfolio.
2. In this paper, AFSA is used to solve the portfolio optimization problem in the investment field, and the algorithm can jump out of the trap of local extreme value in the optimization process, so as to achieve the optimal value. At the same time, the classical test function is used for simulation verification, and a variety of investment decision-making schemes that meet the requirements of investors are obtained. This study broadens the research field of portfolio and AFSA to a certain extent.

## 2. Related Work

As a nonlinear programming problem, the research goal of portfolio problem is to allocate the weight to the individuals of a specific asset set, so as to maximize the total return or minimize the total risk. Nowadays, many scholars use swarm intelligence method to solve this kind of problems. At present, the research and application of AFSA have penetrated into many disciplines and were used to solve practical problems. The algorithm has become a cutting-edge research topic with high research value. Some scholars have studied the application of AFSA in combinatorial optimization, but the research in this field needs to be further deepened.

For solving the nonlinear foreign exchange portfolio multiobjective optimization problem, Neumuller and Rothschild added liquidity conditions and proposed a unified method for solving portfolios [9]. Howton et al. carried out measurement research on the risk of investment portfolio, but there is still strong uncertainty in the measurement of investment return and risk [10]. Vikas et al. proposed the optimal portfolio model under the probability criterion. However, there is no explicit discussion on investment risk, and investment risk is implicitly described by the covariance of returns [5]. Au-Yeung and Chan used a compromise algorithm to solve portfolios with linear transaction costs [11]. Low et al. studied the relative effectiveness of proxy securities, the equilibrium and disequilibrium of portfolio investment, the interaction between investment decision-making and capital structure optimization, and portfolio selection under financing constraints [12]. Bekiros et al. studied a multiperiod portfolio optimization problem and algorithm with feedback control in a fuzzy environment [13]. Sabine finally established a multiobjective nonlinear programming model with transaction fees by adding another objective to generate a multiobjective function of nonlinear programming under the premise of including the objectives of rate of return, risk, liquidity, and so forth [14]. In order to improve the deficiencies of variance model, Eslamloueyan and Jafari introduced conditional value-at-risk to measure asset investment risk and established a multiobjective investment optimization model. Experiments show that the model can improve the ability of investors to manage investment risks [15]. Based on the subjective expected utility theory, Corradin constructed the dynamic prospect theory value objective function model by using the dynamic aversion coefficient and the wealth reference point; at the same time, considering the opportunity constraints to ensure the capital security of investors, the multifrequency vibration mutation particle swarm algorithm was used to analyze the model. Experiments have proved that it has good feasibility in the formulation of practical investment decisions [16]. In order to simplify the covariance matrix, Luque et al. introduced a multifactor tool. Experiments have verified that the method can effectively solve the large-scale investment portfolio problem [17]. Jappelli and Padula reviewed the evolution, improvement, integration with other algorithms, and applications of AFSA in various fields in recent years [18].

The research of these algorithms has played a positive role in promoting the solution of portfolio model. This paper makes a relatively in-depth study on the relevant literature and puts forward the financial market portfolio optimization model based on AFSA. In this paper, the field of vision and step size in traditional AFSA are improved by self-adaptation, and the field of vision and step size are gradually reduced from large to small, so that it can meet the different state needs of algorithm optimization. Try to use standardized and random selection behavior to add variation factors and improve the search strategy of foraging behavior, and then iterate through roulette to select the best of the whole fish school, so as to solve the problem of accuracy and stability. The simulation results show that the algorithm in this paper has good performance and can solve the problem of portfolio optimization.

## 3. Methodology

### 3.1. Financial Market Portfolio

The rational allocation of various assets to best meet the investors' trade-off between risks and benefits is referred to as portfolio management. In finance, portfolio theory is a key asset management theory. During the early stages of portfolio theory development, investors primarily used diversification as an investment strategy, making decisions based on their understanding of market information as well as their historical experience and skills [19]. According to this theory, the risks in portfolio problems decrease as the number of assets in the portfolio decision increases, and a diversified portfolio of assets with very low correlation can effectively reduce the risks. Investing behavior is frequently a long-term process for investors. In general, a portfolio is a research method that allows investors to correctly choose the distribution ratio of capital investment when confronted with multiple capital options in order to maximize profits while minimizing risks. Modern finance and modern portfolio theory are built around the portfolio problem [20]. This problem discusses how to rationally distribute an investor's wealth among various assets in order to achieve the goal of stable and rapid capital growth while also controlling investment risks. Effective portfolio must meet the two following conditions: ① Risk is minimized under the given expected rate of return. ② Under the condition of given risk, the expected rate of return is maximized. All efficient portfolios constitute efficient boundaries or efficient frontiers.

Investment risk is the possibility that the expected investment income will be lost due to the fluctuation of the invested assets as a result of objective factors such as the market environment. The manner in which an investment is made determines the risk of the investment. According to the causes of the risk, we can usually divide the risk into market risk, credit risk, liquidity risk, and operational risk. In a stochastic uncertain investment environment, the return on risky assets is typically assumed to be a random variable. Based on historical asset return data, investors can accurately predict the probability distribution of their future returns. The term “risk” refers to the fact that the rate of return on assets is uncertain [21]. The term “risk-free assets” refers to assets with a guaranteed rate of return. Risk-free assets have at least two characteristics: fixed income and no risk of default. In actual investment activities, portfolio investment, as opposed to single asset investment, can play a role in dispersing overall investment risk, so portfolio investment is a common problem in financial activities. The goal of an investor's investment is to realize consistent and rapid growth of their funds while effectively managing investment risks. During this time, investors must constantly readjust their wealth in order to realize their investment intention, that is, to minimize risks and maximize returns. Any investor wants to maximize his return on investment, but higher returns come with higher risks. The investment decision-making process is a trade-off between yield and risk, which is a decision-making problem with two objectives. The reason for building a portfolio is to reduce the investment risk to a certain extent. Investors can find a trade-off point between the investment income and the corresponding investment risk in the form of portfolio, so as to maximize the total income as much as possible when the investment risk is certain or minimize the investment risk as much as possible when the income is certain.

### 3.2. Portfolio Optimization

The so-called optimization algorithm, in fact, describes a search process or rule, and it is based on a certain idea or mechanism, and through certain search rules and ways, it can obtain the solution to the problem that users want. Combinatorial optimization is one of the optimization problems. It usually refers to the optimization process of finding the optimal arrangement, grouping, sequence, or screening of discrete events through the study of mathematical methods or finding the optimal solution of the objective function from a limited number of feasible solutions. When optimizing the objective function, the objective function with constraints is often involved, so the solution process is complicated. With the development of global economy, the risk of financial market is also increasing. At the same time, with the development of computer technology and the explosive growth of data in solving practical problems, the traditional mathematical solution has been unable to solve practical problems. Therefore, the use of artificial intelligence technology to solve combinatorial optimization problems arises at the historic moment. At present, AFSA has been widely used. In the research field of mathematical theory, some progress has been made in optimizing the objective function by AFSA, and people have paid attention to solving constrained combinatorial optimization problems. AFSA is a new swarm intelligence optimization algorithm based on simulating biological behavior. In the optimization algorithm, the concept of animal autonomy is introduced, and the bottom-up design idea is used, thus forming a new intelligent algorithm.

**Figure 1:**
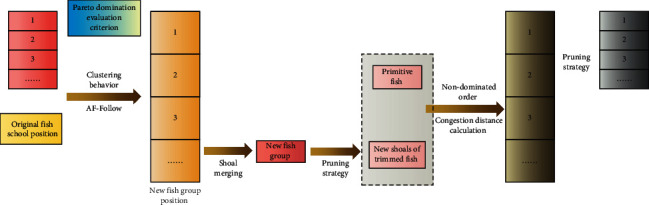
Schematic diagram of the optimization algorithm in this paper.

The intelligent algorithm not only reduces the labor of solving by hand but also lacks the precision of solving by hand. We can quickly find useful information hidden in a large number of data sets, including rules, concepts, patterns, and rules, by combining computer technology and a combinatorial optimization model. Traditional portfolio models are based on probability theory and treat problem uncertainty as a random phenomenon. However, there is a lot of uncertainty in the securities market. Fuzziness is more important than randomness in the field of subjective cognition. The modern portfolio invests in risk diversification and employs quantitative research methods to determine the optimal portfolio investment ratio. The risk of the portfolio decreases as the number of investment types in the portfolio increases. The primary goal of studying portfolio models is to learn how to control the distribution ratio of a portfolio in order to maximize its return. The algorithm is primarily used to rigorously describe a program's iterative steps to solve problems, and it is a generalized general program to solve problems step by step. For an arbitrary given problem, we say it can solve the example if there is such a method that can find the answer to the given problem after a certain step. During the investment process, investors must consider the expected return rate as well as the potential risk rate of purchasing securities. The portfolio model must be optimized in order to provide more objective and optimal decisions. The schematic diagram of optimization algorithm in this paper is shown in Figure 1.

In order to diversify the investment risk and obtain appropriate investment income, investors often use portfolio investment, that is, invest a sum of money in several different securities at the same time. The expression of mean-variance plays a very important role in the construction of portfolio model, providing an objective research tool for rational investors, and is the representative of current portfolio theory. In the mean-variance model, the expected return rate is the expected value of the return rate of the portfolio, and the expected risk refers to the variance of the return rate of the portfolio. Although the mean variance has laid the foundation of modern portfolio theory and has been widely recognized and applied, it still has some limitations. For example: ① Variance or standard deviation cannot determine the direction of income deviation and the possibility of future risks of investment, which are more concerned by investors. ② Generally, the return on assets in the actual market cannot meet the condition that it must conform to the normal distribution, which is a strict premise. ③ The investment portfolio model based on this theory has a large amount of calculation. The main innovation of the statistical analysis method of mean-variance probability is to quantify the income risk in the portfolio problem. Because the income and risk in the investment process are random and uncertain, it is better to use random variables in mathematical statistics for statistical analysis. The mean-variance model assumes that investors are risk-averse. Rational investors always hope to obtain the maximum expected return under the condition of restraining the risk and minimize the investment risk under the condition of restraining the expected return. A portfolio with this nature is called an effective portfolio. Compared with allowing short selling, when using Markowitz mean-variance model to select the optimal investment proportion vector under the condition of not allowing short selling, the opportunity space of portfolio will often be reduced. However, to a certain extent, not allowing short selling can reduce market risks and investors' risks. Therefore, short selling is often not allowed in underdeveloped markets.

### 3.3. Construction of Portfolio Optimization Model in Financial Market Based on AFSA

**Figure 2:**
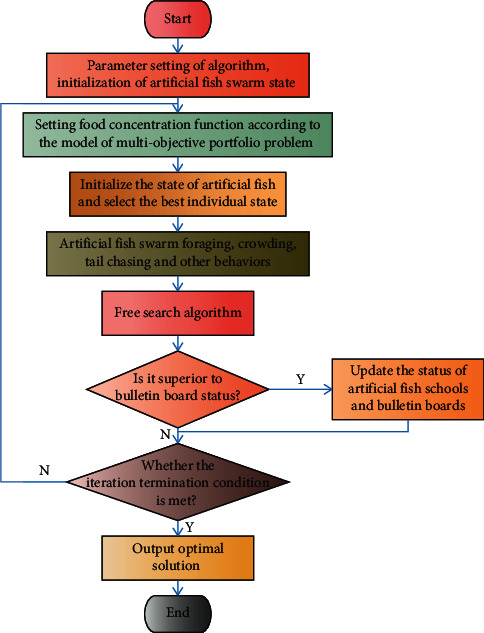
Process of the improved artificial fish free search algorithm.

Artificial fish can realize the mutual conversion of these behaviors at different times through perception of the environment; that is, they can realize their own behavior through behavior evaluation. The food concentration and crowding distance are used to evaluate behavior. Artificial fish continue to move towards areas with high food concentration but are not overcrowded; then choose the best or better behavior, and finally realize the fish swarm convergence. Traditional AFSA has a number of flaws. For example, a fixed field of vision and step size may cause convergence speed to be slow or convergence accuracy to fail, and it may sometimes fall into the local extreme value and fail to reach the global optimum. Furthermore, the algorithm's mathematical foundation is lacking as is the corresponding theoretical analysis. Based on the shortcomings of the traditional AFSA, this section improves the financial market portfolio one by one. The basic strategy for artificial fish movement in this paper is no longer applicable. As a result, this paper proposes a normalization process to address the overflow problem of feasible solutions and employs dynamic vision and step size to improve the search ability of the fish swarm algorithm, avoiding local extremum and increasing optimization accuracy. When a typical transaction cost function is introduced, the model's constraint function becomes nonlinear, and the effective solution set becomes nonconvex and nonconcave, indicating that the search space is irregular. This necessitates using a highly robust algorithm that avoids falling into the local optimum. The flow chart of the improved free search algorithm of artificial fish swarm is shown in Figure 2.

The basic alternate behavior is foraging behavior. If you cannot find a better state than the current one in the process of clustering or rear-end collision, you will do foraging behavior. In foraging behavior, if you cannot find a better state than yourself, you will randomly select a location to update. This will lead to retrogression in the process of optimization. In view of this situation, the solution of this paper is to regard the current solution as a vector, and the next position is no longer obtained randomly, but offset the random angle based on the original direction. The point where AFSA triggers the uniform mutation of the fish school is that when the difference of the global optimal value in the bulletin board for several consecutive times is very small and the number of optimization iterations is not reached, the fish school begins to produce uniform mutation and generates a new fish school state. In this paper, the fitness of individuals is measured by an evaluation function based on ranking. Evaluation function is used to set a probability for each chromosome in the population, so that the probability of this chromosome being selected is proportional to its adaptability to other chromosomes in the population. That is, through roulette, the chromosome with strong adaptability is selected to have a greater chance of producing offspring. In the process of applying the algorithm, this paper tries to reduce the number of individuals and scale of the fish school as much as possible on the premise of ensuring the convergence of the stable algorithm. In this algorithm, with more fish swarm individuals set, the frequency of information exchange between artificial fish individuals will increase, and the convergence speed of the algorithm will be accelerated, so as to better jump out of the local extreme value. However, the amount of calculation and optimization time in each optimization iteration of the algorithm are also increased. Therefore, reasonably specifying the size of fish swarm is the key to improve the efficiency of the algorithm. In the past, artificial fish swarms did not consider individuals with poor search effect in the population. In this regard, this paper uses roulette to screen according to the fitness function value of each individual and seeks individuals with strong ability to iterate to the next generation with a large probability. AFSA embodies the natural law of survival of the fittest for all fish groups throughout the evolution of animals. Suppose that the *i*-th individual *x*_*i*_ in the artificial fish swarm is given a probability *P*_*b*_ of mutation; randomly select the subelement *x*_*ij*_ at position *j* in *x*_*i*_ to mutate it.

(1) $$\begin{array}{c} \text{if }r\mathrm{and}<P_{b},\\ x_{ij}=r\mathrm{and}\\ x_{i/\text{next}}=\text{norm}\left(x_{i/\text{next}}\right). \end{array}$$

The basic fish swarm algorithm selects and randomly generates a new solution after it is not optimized, which may lead to the regression of the solution. This paper uses the following formula to deal with it:

(2) $$\begin{array}{c} x_{i/\text{next}}=\left\{\begin{array}{cc} x_{i}+\left(x_{v}-x_{i}\right)*\text{dis}\left(x_{v}-x_{i}\right); & \text{if }Y_{i}>Y,\\ \text{ob}\left(x_{i}\right); & \text{else if }Y_{ob\left(x_{i}\right)}>Y,\\ x_{i}; & \text{else,} \end{array}\right.\\ x_{i/\text{next}}=\text{norm}\left(x_{i/\text{next}}\right).. \end{array}$$

In the above formula, *ob*(*x*_*i*_) is the vector obtained by taking *x*_*i*_ as a direction vector and offsetting it by a certain angle. According to the fitness function value, the artificial fish individuals with strong optimization ability are screened out by roulette to enter the next generation.

(3) $$\begin{array}{c} \text{fish}-{\text{swarm}}_{i/\text{next}}=\text{roullete}\left(\text{fish}-{\text{swarm}}_{i}\right). \end{array}$$

Suppose that investors consider four decision-making factors: wealth, risk, skewness, and kurtosis of the portfolio terminal. It is required to maximize the terminal wealth and cumulative skewness of the portfolio and minimize the cumulative risk and kurtosis of the portfolio. According to the uniform mutation operator in genetic algorithm, the mutation characteristics are combined with AFSA, so that the fish swarm algorithm has the same mutation ability, and it can avoid falling into local extremum, which is more conducive to the fish swarm reaching the global optimum in the shortest time. A single error index cannot fully evaluate the prediction effect of the model. Therefore, MAE (mean absolute error), RMSE (root mean square error), and CTR (correct trend rate) are introduced to evaluate the prediction accuracy, and the running time of the algorithm is used to evaluate the convergence speed. Its calculation formula is as follows:

(4) $$\begin{array}{c} \text{MAE}=\frac{\sum_{i=1}^{n}\left|y_{i}^{\prime }-y_{i}\right|}{n},\\ \text{RMSE}=\sqrt{\frac{1}{n}\sum_{i=1}^{n}\left(y_{i}^{\prime }-y_{i}\right)},n\\ \text{CTR}=\frac{\sum_{i=1}^{n}{\text{CTR}}_{i}}{n}. \end{array}$$

In the above formula, *y*_*i*_ and *R*_*i*_ are the income of the asset on the current day and the previous day, respectively, and *i* is the return of the asset on the *i* day; namely,

(5) $$\begin{array}{c} R_{i}=\frac{y_{i}-y_{i}-1}{y_{i}-1}\;i=2,\ldots ,\;n=220. \end{array}$$

The expected rate of return is

(6) $$\begin{array}{c} \frac{y_{i}^{\prime }-y_{i}-1}{y_{i}-1}. \end{array}$$

Combined with the portfolio model constraint function studied in this paper, an outlier penalty function is introduced:

(7) $$\begin{array}{c} \max F\left(x\right)=\delta \sum_{i=1}^{n}\left(Kx_{i}R_{i}-\prod_{i}\right)+\left(1-\delta \right)\left\{\underset{1\leq i\leq n}{\max}\left\{Kx_{i}S_{i}\right\}\right\}-\gamma \left(\sum_{i=1}^{n}x_{i}-1\right). \end{array}$$

In the above formula, *γ*=8^*k*^(*k*=1,2,3,…).

The perception of artificial fish's range or distance is known as vision. The algorithm's convergence speed is generally determined by the value of the visual field. As a result, in order to observe the search space more thoroughly, the sensing range should be expanded, making it easier for artificial fish to locate and converge on the global extremum. For multiobjective decision-making problems, there is usually no optimal solution in the traditional sense due to the incommensurability and contradiction of objectives. A common method for resolving this type of problem is to convert the multiobjective decision-making problem into the corresponding single-objective decision-making problem. The traditional AFSA is limited in its search for the optimal solution of the objective function in the solution space of its research problem because the vision and step size of the fish swarm algorithm are typically fixed and artificially set before the experiment begins. In AFSA, each parameter's setting is not sensitive, and there is no strict theoretical basis for selecting the optimal parameter. The algorithm is extremely robust. However, because the algorithm is random, the value of each parameter has some influence on the algorithm's convergence process and optimization results. In this paper, adaptive field of vision and step size are used, which means that, in the early stage of convergence, a larger field of vision and step size are used so that it can be quickly positioned in the global optimal state; in the late stage of convergence, a smaller field of vision and step size are used to improve the algorithm's convergence accuracy.

## 4. Result Analysis and Discussion

The experimental data of this paper are the trading data of 25 stocks from the stock market for 450 trading days. The rate of return is the relative average rate of return. The data sent by the sample can minimize the interference caused by relevant errors in the experiment and can accurately demonstrate the performance of the algorithm. In this chapter, Matlab software is used for experiments. Run a 64-bit operating system with 16 GB of memory. Before the simulation, initialize the parameters. The experimental parameters for testing the performance of the algorithm are set as follows: the number of artificial fish school is 100; the initial visual field size is 1; the maximum number of attempts is 80; the mutation operator is 0.05; and the visual field distance is 1; the maximum number of iterations is 100. CTR, MAE, and RMSE experiments are carried out with different algorithms, and the results of three indexes are shown in Table 1.

**Figure 3:**
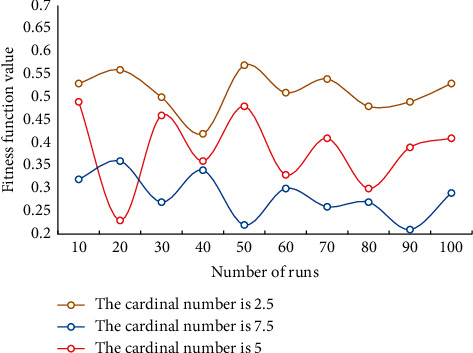
Influence of cardinality constraint on optimal value.

**Figure 4:**
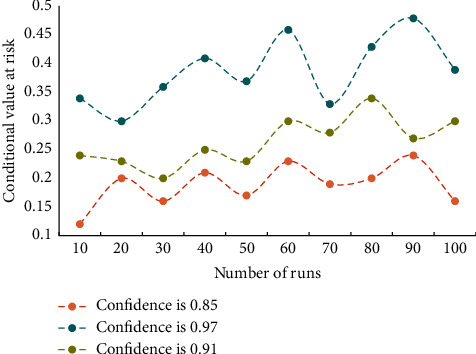
The effect of confidence on the optimal value.

It can be seen from the data in the table that the MAE and RMSE of the algorithm in this section are lower than those of other algorithms, and the CTR of the algorithm in this paper is the highest. In order to verify the effectiveness of this algorithm, we run the algorithm 100 times and test the influence of cardinality constraint and confidence value on the optimal value of this paper. The influence of cardinality constraint on the optimal value is shown in Figure 3. The influence of confidence on the optimal value is shown in Figure 4.

**Figure 5:**
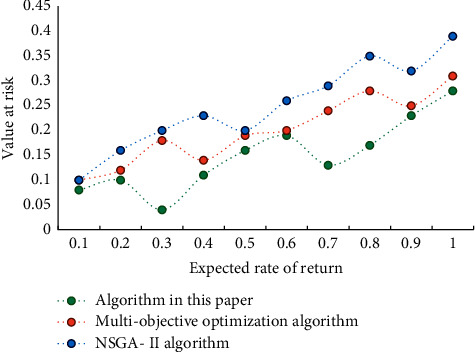
Comparison of pareto frontier distributions for different algorithms.

Figure 3 shows that the overall trend is that the value of conditional risk value decreases with the increase of base constraint. This is in line with the principle that diversified investment can reduce nonmarket risks. As can be seen from Figure 4, when the expected return is constant, with the increase of confidence value, the conditional risk value also increases correspondingly. This shows that investors' aversion to risks has increased.  Figure 5 shows the comparison results of Pareto frontier distribution of different algorithms.

It can be seen from Figure 5 that the result obtained by the improved AFSA has the lowest risk under the same expected rate of return, and it approaches the optimal solution of linear programming well, and a group of Pareto optimal solutions are found. The improved AFSA and the traditional AFSA were tested by the test function, and the test was repeated 50 times in an independent environment. The test results are shown in Table 2.

**Figure 6:**
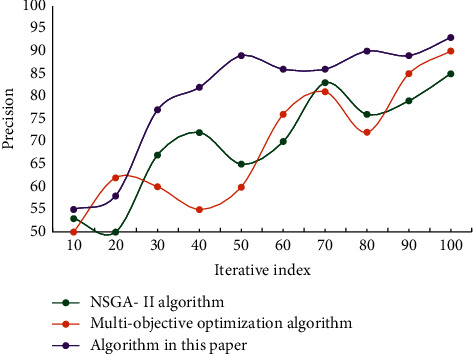
Comparison of the accuracy results of different models.

It can be seen from Table 2 that the improved AFSA has stronger overall optimization solution ability and better robustness than the traditional AFSA. The success rate of the improved algorithm in optimizing the test function has been greatly improved. To verify the effectiveness and reliability of the improved method, the precision experiment of the model is carried out, and the results are shown in Figure 6.

**Figure 7:**
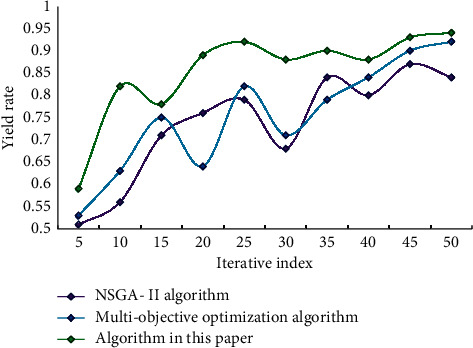
Model performance test.

It can be seen that the accuracy of the improved model is obviously higher than those of the other two models. The accuracy of this model can reach 93.56%, which is 8.43% higher than that of NSGA-II model and 3.76% higher than that of multiobjective optimization model. This result verifies the effectiveness and reliability of the improved method in this paper. Using this model, NSGA-II model, and multiobjective optimization model to test the performance, the results are shown in Figure 7.

From the data analysis in Figure 7, it can be concluded that most of the total returns of the optimized portfolio model in this paper are higher than those of other models. It can obtain a better portfolio decision scheme, make the investment income as large as possible, and minimize the risk and provide investors with a certain investment direction. This shows that the portfolio model in this paper is effective, and its optimal investment strategy can be applied to actual investment.

The improved AFSA shows more reliable convergence performance than the traditional AFSA because it refers to the solution strategy with strong theoretical basis in genetic algorithm. At the same time, due to the inherent advantages of AFSA, it also overcomes the problems such as the sensitivity of genetic algorithm to initial value. The experimental results show that it can eliminate the local extremum and finally obtain the global optimal state, which effectively solves the problem of low convergence accuracy of the algorithm optimization problem.

## 5. Conclusions

Market competition is prevalent at the moment, and it is becoming increasingly intense and diverse. Investors should be prepared for potential risks while also considering the numerous advantages that investment can provide. As a result, the current research focus is on how to select an investment strategy to maximize profits. The related literature are studied fairly thoroughly in this paper, and a financial market portfolio optimization model based on AFSA is built. Simultaneously, the corresponding improvement methods are proposed to address the shortcomings of traditional AFSA, such as low convergence accuracy, slow speed, and the tendency to fall into local extremum. Combining AFSA with a free search algorithm improves the optimization performance of traditional AFSA and, as a result, strengthens the overall algorithm's global ergodicity and local delicacy. According to empirical evidence, this model's accuracy can reach 93.56 percent, which is 8.43 percent higher than that of the NSGA-II model and 3.76 percent higher than that of the multiobjective optimization model. Simultaneously, the Pareto frontier obtained by improving AFSA is more uniform and diverse, indicating that the Pareto optimal solution is of higher quality. This demonstrates the improved algorithm's effectiveness and applicability. The optimal decision optimized by the improved fish swarm algorithm has a higher investment benefit, a higher expected return, and a lower risk of portfolio investment. This is beneficial to the prosperity and development of the securities trading market because it promotes investors' investment and trading activities. According to the findings of the preceding research, AFSA is a viable option for addressing the portfolio problem. AFSA has significant development potential, which merits our thorough and extensive investigation. By improving and perfecting it, AFSA is expected to have a better application prospect. The improved AFSA in this paper achieved good optimization results, but the index selection of the investment portfolio is not detailed and comprehensive enough, and how to improve the stability of AFSA performance without sacrificing efficiency will be researched further in the future.

## Figures and Tables

**Table 1:** Comparison of the results of three indicators of different algorithms.

Algorithm	CTR	MAE	RMSE
Support vector machine algorithm	0.878	0.501	0.649
Multiobjective optimization algorithm	0.623	0.279	0.387
Particle swarm optimization	0.807	0.328	0.416
NSGA-II algorithm	0.745	0.496	0.485
Basic fish swarm algorithm	0.815	0.287	0.389
Algorithm in this paper	0.647	0.213	0.347

**Table 2:** Optimization results of test functions for different algorithms.

Index	Test 1		Test 2	
	Traditional AFSA	Improved AFSA	Traditional AFSA	Improved AFSA
Optimal solution	0.994	1	164.72	174.97
Worst solution	0.978	1	163.57	174.92
Average function value	0.983	1	163.98	174.96
Average number of convergence iterations	20	8	18	12
Success rate (%)	74.5	99.6	82.6	99.2

## Data Availability

The data used to support the findings of this study are available from the author upon request.
